# Antitumorigenic Effects of Inhibiting Ephrin Receptor Kinase Signaling by GLPG1790 against Colorectal Cancer Cell Lines *In Vitro* and *In Vivo*

**DOI:** 10.1155/2020/9342732

**Published:** 2020-02-27

**Authors:** Alessandro Colapietro, Giovanni Luca Gravina, Francesco Petragnano, Irene Fasciani, Bianca Maria Scicchitano, Filip Beirinckx, Philippe Pujuguet, Laurent Saniere, Ellen Van der Aar, Daniela Musio, Francesca De Felice, Vincenzo Mattei, Stefano Martellucci, Roberto Maggio, Vincenzo Tombolini, Claudio Festuccia, Francesco Marampon

**Affiliations:** ^1^Department of Biotechnological and Applied Clinical Sciences, Laboratory of Radiobiology, University of L'Aquila, L'Aquila, Italy; ^2^Department of Biotechnological and Applied Clinical Sciences, Laboratory of Pharmacology, University of L'Aquila, L'Aquila, Italy; ^3^Istituto di Istologia ed Embriologia, Università Cattolica del Sacro Cuore, Fondazione Policlinico Universitario Agostino Gemelli, Largo Francesco Vito 1-00168, Roma, Italy; ^4^Galapagos NV, Mechelen, Belgium; ^5^Galapagos SASU, Romainville, France; ^6^Department of Radiotherapy, Hospital Umberto I “Sapienza”, University of Rome, Rome, Italy; ^7^Laboratory of Experimental Medicine and Enviromental Pathology, University Hub “Sabina Univeristas”, Rieti, Italy

## Abstract

Erythropoietin-producing hepatocellular receptors (Eph) promote the onset and sustain the progression of cancers such as colorectal cancer (CRC), in which the A2 subtype of Eph receptor expression has been shown to correlate with a poor prognosis and has been identified as a promising therapeutic target. Herein, we investigated, *in vitro* and *in vivo*, the effects of treatment with GLPG1790, a potent pan-Eph inhibitor. The small molecule has selective activity against the EphA2 isoform in human HCT116 and HCT15 CRC cell lines expressing a constitutively active form of RAS concurrently with a wild-type or mutant form of p53, respectively. GLPG1790 reduced EPHA2 phosphorylation/activation and induced G_1_/S cell-cycle growth arrest by downregulating the expression of cyclin E and PCNA, while upregulating p21^Waf1/Cip1^ and p27^Cip/Kip^. The inhibition of ephrin signaling induced quiescence in HCT15 and senescence in HCT116 cells. While investigating the role of CRC-related, pro-oncogenic p53 and RAS pathways, we found that GLPG1790 upregulated p53 expression and that silencing p53 or inhibiting RAS (human rat sarcoma)/ERKs (extracellular signal-regulated kinase) signaling restrained the ability of GLPG1790 to induce senescence in HCT116 cells. On the other hand, HCT15 silencing of p53 predisposed cells to GLPG1790-induced senescence, whilst no effects of ERK inhibition were observed. Finally, GLPG1790 hindered the epithelial-mesenchymal transition, reduced the migratory capacities of CRC, and affected tumor formation in xenograft models *in vivo* more efficiently using HCT116 than HCT15 for xenografts. Taken together, our data suggest the therapeutic potential of GLPG1790 as a signal transduction-based therapeutic strategy in to treat CRC.

## 1. Introduction

Colorectal cancer (CRC) is the third and second most commonly occurring cancer in men and in women, respectively, with 1 million of new cases diagnosed every year [1]. CRC develops through different stages and is promoted by the accumulation of acquired or inherited genetic mutations that eventually promote malignant evolution [2]. In this regard, mutation of adenomatous polyposis coli (APC), Kirsten rat sarcoma viral oncogene homolog (KRAS), and tumor protein 53 (TP53 or p53) have been extensively investigated and have been shown to play a key role in the progression of CRC [3]. However, CRC cells comprised complex genetic traits, and the role of additional mutations or aberrant expression of additional genes remains unknown.

The erythropoietin-producing hepatocellular carcinoma receptors (Eph) constitute the largest family of receptor tyrosine kinases (RTKs). The family comprised sixteen total receptors divided into either A- or B-subclasses. The EphA group consists of EphA1 to EphA10 receptors, and the EphB group comprised six receptors named EphB1 to EphB6. Eph proteins bind membrane-bound ligands named ephrins generating a very complex signaling network. Eph receptors and ephrins expressed in different cells interact in *trans* to activate bidirectional signaling cascades from Eph and ephrin, which have been called “*forward*” and “*reverse*” signals, respectively. Eph and ephrins coexpressed in the same cell interact in *cis*, a phenomenon that inhibits *trans* signaling [4, 5].

Eph regulates several biological processes through different kinase-mediated *forward* and *reverse* pathways that include small GTPases of Rho members, RAS, focal adhesion kinase (FAK), the PI3 kinase pathway (PI3K), the Jak/Stat pathway, and Src [4, 5]. Because of their critical role in regulating several physiological processes, the aberrant expression of Ephs and ephrins has been reported in many human tumors including CRC [6, 7]. Aberrant expression of Ephs and ephrins has also been shown to promote cancer cell growth, migration, and invasion through the disruption of multiple molecular mechanisms [5–7]. Several studies shown that significant upregulation of EphA1, EphA2, EphA3, EphA4, EphB2, and EphB4 occurs throughout CRC progression [7, 8], and EphA2 has been shown to be a marker for advanced disease and a poor prognosis and is a critical therapeutic target [9].

We have recently developed a preclinical, orally bioavailable small molecule named GLPG1790 that is able to inhibit, at nanomolar concentrations, the activity of various Eph receptors, with a particularly strong efficiency versus EphA2 [10]. The molecule to efficiently diminishes the phenotypic transformation of several breast, rhabdomyosarcoma, and glioblastoma cancer cell lines, both *in vitro* and *in vivo* [10–12], and our recent preliminary data suggest a potential therapeutic role for GLPG1790 also in treating CRC [13]. The present study has investigated the therapeutic efficiency of GLPG1790 against HCT116 and HCT15 CRC cell lines, both expressing a mutated form of KRAS in addition to either a wild-type p53 protein (p53^WT^) or mutated p53 (p53^MT^), respectively [14]. Herein, GLPG1790 significantly inhibited tumor cell growth both *in vitro* and *in vivo*, where treatment-induced quiescence or senescence in a cell type-dependent manner and affected the epithelial-mesenchymal transition program. Interestingly, we found that the mutational status of p53, along with RAS (human rat sarcoma)/ERKs (extracellular signal-regulated kinase) signaling, affected GLPG1790-mediated anticancer activity that was higher in p53^WT^ expressing cells. Thus, data collected here support the idea that targeting Eph may offer a new means of developing novel anticancer strategies against CRC.

## 2. Materials and Methods

### 2.1. Cell Cultures, Viability, Proliferation, Wound-Healing Assay, *β*-Galactosidase Activity Assay, and FACS Analysis

Human colorectal cancer cells (HCT116 and HCT15) were purchased from American Type Culture Collection (Rockville, MD, USA). Colorectal cancer cells were cultured in DMEM (Dulbecco's modified medium) supplemented with 10% FBS and 1% glutamine and 1% streptomycin or penicillin. The cells were incubated at 37°C and 5% CO_2_, with humidity. Evaluation of cell viability (IC_50_) and proliferation, wound-healing assays, FACS analysis, and *β*-galactosidase assays were performed as previously described [12, 15].

### 2.2. Transient Transfection

Transient transfection of cells was performed as previously described [16]. Briefly, HCT116 and HCT15 cells were seeded at 50–60% confluence in 6-well plates. Small interfering RNA (siRNA) against human p53 (p53-siRNA, sc-29435; Santa Cruz Biotechnology, Dallas, TX) or siRNA-negative control (CTR-siRNA, sc-37007; Santa Cruz Biotechnology, Dallas, TX) were combined with RNAiMAX (Invitrogen) and used at 60 nM final concentration. The p53-siRNA exists as a pool of three target-specific, 19–25 nt siRNAs designed to specifically knock down the targeted gene.

### 2.3. Western Blotting

Western blot analyses were performed as previously described [17, 18] using the following primary antibodies: EphA2 (C-3), p27^Cip/Kip^ (F-8), Survivin (D-8), E-cadherin (G-10), PCNA (FL-261), beta-galactosidase (B-12), p21^Waf1/Cip1^ (C-19), p16 (F-12), p53 (DO-1), phospho-ERK1/2 (Tyr204) (E-4), CDK4 (DCS-35), cyclin D1 (M-20), cyclin E (HE12), phospho-PAK4 (Thr423), PAK4 (B3), Ku70 (H-308), Ku86 (H-300), ERK1/2 (C-14) (Santa Cruz Biotechnology, CA, USA), *α*-tubulin (TU-02), phospho-p38 (Thr180/Tyr182) (9211), phospho-EPHA2 (Ser897) (D9A1) by Cell Signaling Technology (Danvers, MA), ZO-1 (D6L1E), and Snail (L70G2) (Cell Signaling Technology, Leiden, The Netherlands). Samples were incubated with appropriate horseradish peroxidase (HRP)-conjugated secondary antibodies (EuroClone, MI, Italy) for 1 h at room temperature. Protein signals were detected using a Western Bright ECL kit (Advansta, Menlo Park, CA) according to the manufacturer's instructions and visualized using a ChemiDoc XRS+ (Bio-Rad, Hercules, CA). Densitometry was performed to quantify changes in protein expression using the Image Lab5.1 software (Bio-Rad) [19, 20].

### 2.4. *In Vivo* Study

Forty, six-week-old, female CD1 nu/nu mice (purchased by Charles River; Milan, Italy) were used for the *in vivo* studies in accordance with the guidelines of our institute (University of L'Aquila, Medical School and Science and Technology School Board Regulations, complying with the Italian government regulation n.116 January 27, 1992, for the use of laboratory animals). 20 animals were inoculated in the flank subcutaneously with an HCT116 or HCT15 suspension composed of 1 × 10^6^ cells in 100 *μ*l of DMED without FBS matrigel. Tumors were allowed to grow for 10 days after inoculation, until masses reached the volume of about 0.2–0.3 cm^3^, which is considered adequate for animal randomization. Then, mice were separated into two groups: a control group and one treated with GLPG1790 (30 mg/kg body weight, five days/week, for four weeks) using a dosage defined by previous pharmacological investigations executed by Galapagos. The following parameters were used to quantify the antitumor effects of different treatments: tumor volume, measured throughout the experiment; tumor weight, measured at the end of the experiment; and tumor progression (TP), defined as a tumor volume increase of greater than 50% with respect to the baseline. Tumor size was measured with calipers, and the TP of any single tumor was calculated. TP data were used to generate Kaplan–Meier curves to describe progression. This method of analysis reduces both intersubject variability resulting from differences in engraftment efficacy, as well as intrasubject variability.

### 2.5. Statistical Methods

Continuous variables were summarized as the standard error of the mean (SEM). For continuous variables, statistical comparisons between control and treated groups were established by carrying out one-way ANOVA tests or the Student's *t*-test (in cases where there were two independent groups). Dichotomous variables were summarized by absolute and/or relative frequencies. For dichotomous variables, statistical comparisons between control and treated groups were established by carrying out Fisher's exact test. For multiple comparisons, the level of significance was corrected by multiplying the *P* value by the number of comparisons performed (*n*) according to the Bonferroni correction. All tests were two-sided and were determined by Monte Carlo significance. *P* values less than 0.05 were considered statistically significant. All statistical analyses were performed using the SPSS^®^ statistical analysis software package, version 10.0 [21].

## 3. Results

### 3.1. GLPG1790 Induces G1/S Cell-Cycle Growth Arrest by Promoting Quiescence of HCT15 and Senescence Status HCT116 Cells

**Figure 1:**
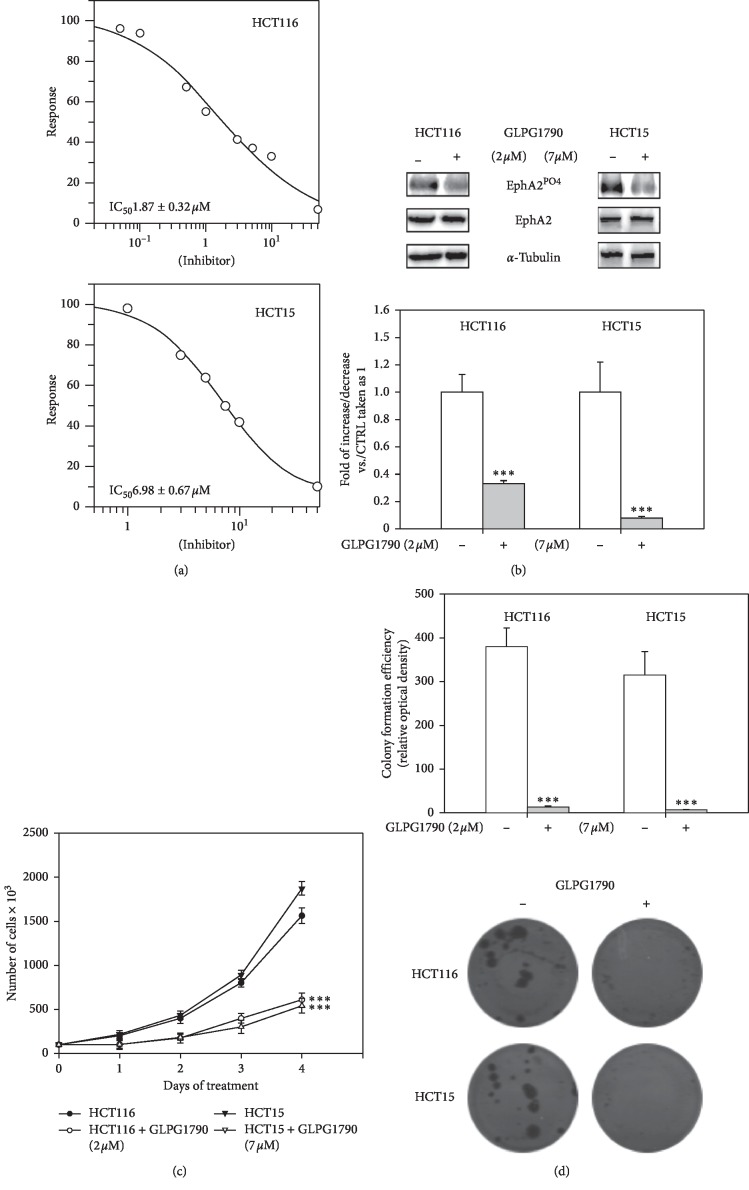
GLPG1790 decreases CRC cell viability, growth potential and anchorage-dependent clonogenic capacity. (a) Dose-dependent effect of GLPG1790 on viability of HCT116 and HCT15 cells after 48 h of treatment has been shown. Cell viability was measured using an MTT assay. Results are representative of three independent experiments ± SD. (b) Cell lysates from HCT116 and HCT15 cells that were either untreated (DMSO) (−) or treated (+) with GLPG1790 (IC_50_) for 24 h were analyzed via immunoblotting with specific antibodies for indicated proteins. *α*-Tubulin expression is a sample loading control. Densitometric analysis of four independent experiments has been reported below the blots (^*∗∗∗*^*P* < 0.001 vs. control). (c) HCT116 and HCT15 cells, grown in adherent conditions, were treated with GLPG1790 (IC_50_) for the indicated periods. The number of the cells was obtained using the trypan blue dye exclusion test. Results represent the mean values ± SD of four independent experiments (^*∗∗∗*^*P* < 0.001 vs. control). (d) Either untreated (DMSO) or GLPG1790-treated HCT116 and HCT15 cells were seeded at low concentration in an anchorage-dependent condition. Colonies were photographed and counted after 14 days of treatment. Results represent the mean values ± SD of three independent experiments (^*∗∗∗*^*P* < 0.001 vs. control).

**Figure 2:**
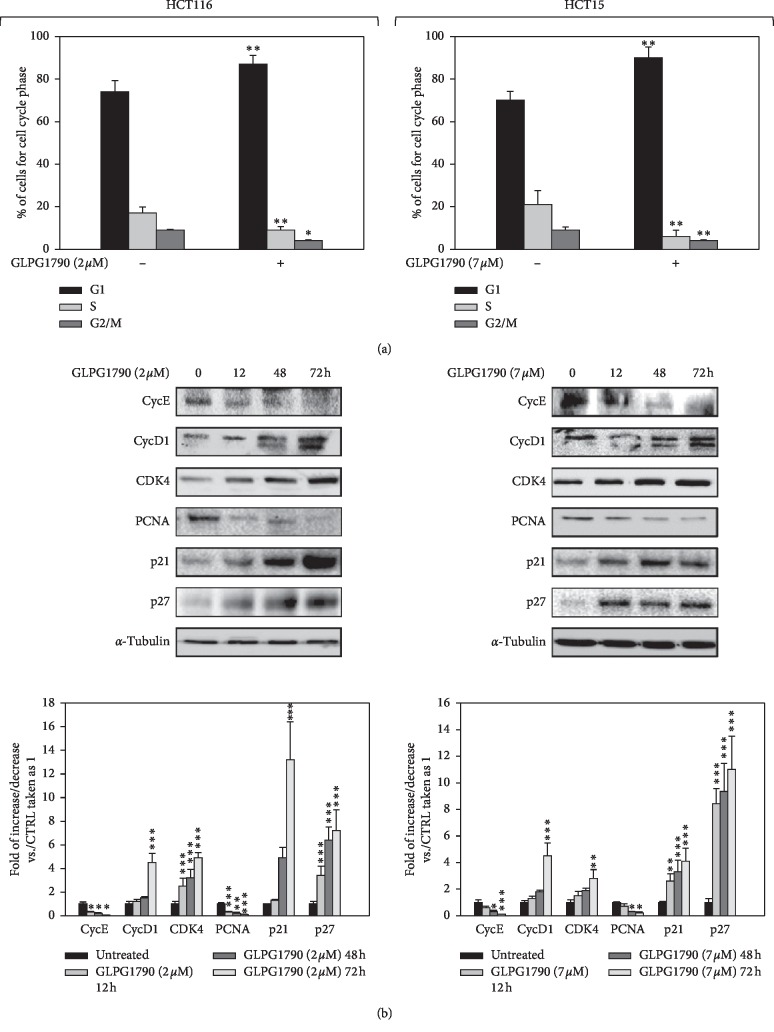
GLPG1790 induces growth arrest in the G1/S phase of the cell cycle and modulates related cell-cycle proteins. (a) FACS analysis performed on HCT116 and HCT15 cells that had been treated with either DMSO (negative control) or GLPG1790 (IC50) for 24 h. Histograms showing the percentage of cell in each cell-cycle phase for HCT116 and HCT15 cells ± GLPG1790. Results represent the mean value of four independent experiments (^*∗*^*P* < 0.05 or ^*∗∗*^*P* < 0.01 vs. control). (b) Cell lysates from HCT116 and HCT15 cells ± GLPG1790 (IC_50_) at the indicated times were analyzed by immunoblotting with specific antibodies for indicated proteins. *α*-Tubulin expression acted as a loading control for samples. Densitometric analysis of three independent experiments has been reported below the blots (^*∗*^*P* < 0.05, ^*∗∗*^*P* < 0.01 or ^*∗∗∗*^*P* < 0.001 vs. control).

**Figure 3:**
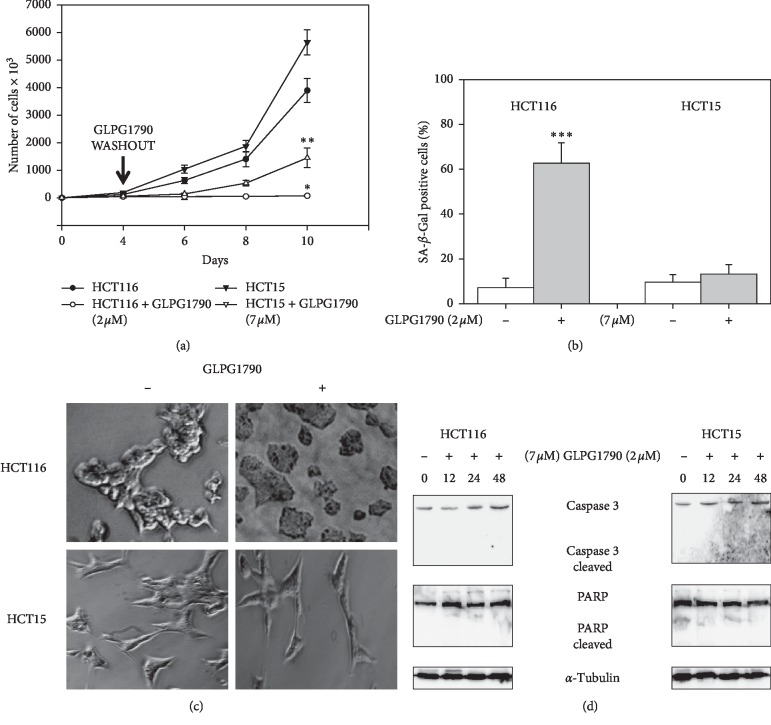
GLPG1790 induces quiescence in HCT15 and senescence in HCT116 cells, depending on the expression of p53. (a) HCT116 and HCT15 cells were treated with 2 *μ*M and 7 *μ*M GLPG1790, respectively, for 4 days before GLPG1790 was removed from the cells. Cell counts were performed 2, 6, and 8 days after the removal of GLPG1790 (^*∗∗*^*P* < 0.005 vs. 4 days of treatment with GLPG1790). (b) *β*-Galactosidase activity was assessed using ELISA after 4 days of GLPG1790 treatment (^*∗∗∗*^*P* < 0.001 vs. untreated). (c) Cellular morphology of HCT116 and HCT15 cells, respectively, treated with 2 *μ*M and 7 *μ*M GLPG1790 was analyzed under light microscope at 20x magnification at 4 days after treatment. (d) Cell lysates from HCT116 and HCT15 cells that were either untreated (DMSO) (−) or treated (+) with GLPG1790 (IC_50_) for 12, 24, and 48 h were analyzed via immunoblotting with specific antibodies for indicated proteins. *α*-Tubulin expression is a sample loading control. Densitometric analysis of four independent experiments has been reported below the blots (^*∗∗∗*^*P* < 0.001 vs. control).

The dose of GLPG1790 able to affect 50% of CRC cell viability (IC_50_) was investigated. After two days of treatment of HCT116 and HCT15 using 0.1–10 *μ*M GLPG1790 produced IC_50_ values of 1.87 ± 0.32 *μ*M and 6.98 ± 0.67 *μ*M, respectively (Figure 1(a)). 24 hours after treating cells with a dosage equal to the IC_50_ of each cell type, GLPG1790 significantly reduced EphA2 phosphorylation/activation by 71 ± 9% in HCT116 and 90.4 ± 6.2% in HCT15 cells (Figure 1(b)). The effect of GLPG1790 on the proliferation rate of cells (Figure 1(c)) and the ability of cells to form colonies (Figure 1(d)) were also investigated. Four days of treatment with GLPG1790 reduced the proliferation rate of CRC cells by 96.5 ± 13.3% in HCT116 and 98.2 ± 18.4% in HCT15 cells. After 14 days of treatment, colonies-forming capacity of HCT116 and HCT15 cells was reduced 66 ± 3.1% and 66 ± 5.2%, respectively. Flow cytometry showed that treating cells with GLPG1790 (IC_50_) for 24 hours significantly reduced DNA replication (S phase) by primarily arresting cells in the G_1_ phase of the cell cycle (Figure 2(a)). Consistent with the G_1_/S arrest, GLPG1790 decreased expression levels of cyclin E (CycE) and PCNA whilst it increased p21^Waf1/Cip1^ and p27^Cip/Kip^ protein expression in both cell lines (Figure 2(b)). Unexpectedly, treatment upregulated the cyclin D1 (CycD1) and CDK4 protein expression both in HCT116 and HCT15 cell lines (Figure 2(b)). Therefore, investigating whether the antiproliferative effects of GLPG1790 were reversible or not, CRC cell lines were transiently treated for 4 days with GLPG1790 (IC_50_). As shown in Figure 3(a), removing GLPG1790 restored the ability of HCT15 to proliferate. This was contrary to effects observed after removing GLPG1790 from HCT116 cells, which remained persistently arrested (Figure 3(a)). Notably, after four days of treatment, *β*-galactosidase activity (Figure 3(b)) and large morphology (Figure 3(c)), widely used as markers of senescent cells, were significantly increased in HCT116 but not in HCT15 cells. GLPG1790 did not induce apoptotic cell death as suggested by the inability of treatment to increase the active (cleaved) form of caspase 3 and PARP (Figure 3(d)). Taken together, these results suggest that the inhibition of EPH signaling is able to affect CRC-transformed phenotypes by inducing either quiescence or senescence, but not cell death, in a cell type-dependent manner.

### 3.2. p53 Mutational Status Participate in Mediating GLPG1790-Induced Senescence Differently than RAS/ERK Signaling

**Figure 4:**
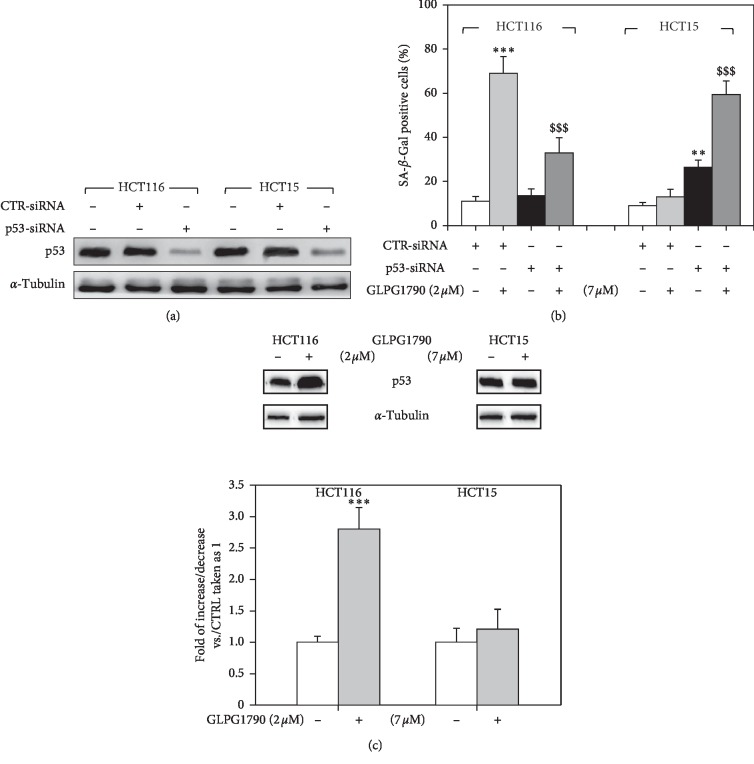
p53 mutational status participate in mediating GLPG1790-induced senescence. (a) Cell lysates from HCT116 and HCT15 expressing p53-siRNA were analyzed by immunoblot 36 h after transfection using specific antibodies for indicated proteins. *α*-Tubulin expression indicates sample loading. (b) *β*-Galactosidase activity was assessed by ELISA. HCT116 and HCT15 p53-silenced cells were treated with GLPG1790 for 36 h (^*∗∗∗*^*P* < 0.001, ^*∗∗*^*P* < 0.005 vs. untreated cells, ^§§§^*P* < 0.001 vs. CTR-siRNA + GLPG1790). (c) Cell lysates from HCT116 and HCT15 cells that were either untreated (DMSO) (−) or treated (+) with GLPG1790 (IC_50_) for 36 h were analyzed via immunoblotting with specific antibodies for indicated proteins. *α*-Tubulin expression is a sample loading control.

**Figure 5:**
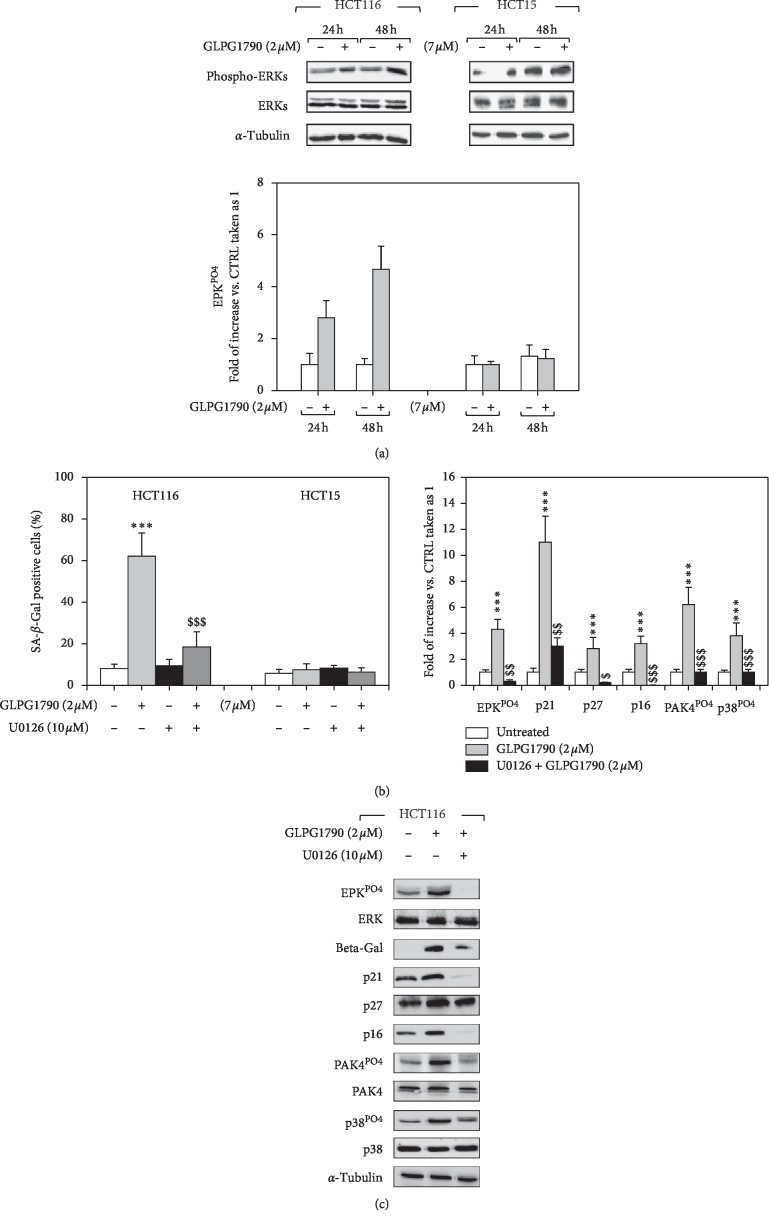
GLPG1790 induces senescence in HCT116 cells via the activation of ERK signaling. (a) Cell lysates from HCT116 and HCT15 cells treated with 2 *μ*M and 7 *μ*M GLPG1790, respectively, for 24 or 48 h were analyzed using immunoblotting with specific antibodies for indicated proteins. *α*-Tubulin expression functions as a loading control. (b) *β*-Galactosidase activity was assessed by ELISA using HCT116 and HCT15 cells that had been cotreated with U0126 (10 *μ*M) and GLPG1790, 2 *μ*M or 7 *μ*M, respectively, for 36 h (^*∗∗∗*^*P* < 0.001 vs. untreated, ^§§§^*P* < 0.001 vs. GLPG1790). (c) Cell lysates from HCT116 and HCT15 cells cotreated with U0126 (10 *μ*M) and GLPG1790, 2 *μ*M or 7 *μ*M, respectively, for 36 h were analyzed by immunoblotting with specific antibodies for indicated proteins. *α*-Tubulin expression functions as a loading control.

The roles of p53 and RAS, both key players in the regulation of senescence of CRC cells [22, 23], were investigated. HCT116 and HCT15 cell lines were transfected with specific small interfering RNAs (siRNAs) directed against p53 mRNA (p53-siRNA), while a sequence against a *C. Elegans* gene (CTR-siRNA) was used as a negative control (Figure 4(a)). Western blotting analysis, performed 36 hours after transfection, revealed that p53 protein levels were specifically reduced in p53-siRNA-transfected cells (Figure 4(a)). 36 hours after transfection, cells were treated with GLPG1790 (IC_50_), and *β*-galactosidase activity (Figure 4(b)) was assessed. As shown in Figures 4(b) and 4(c), silencing p53 diminished the ability of GLPG1790 to promote *β*-galactosidase activity 54.2 ± 7.1% in HCT116 (Figure 4(b)). However, *β*-galactosidase activity was enhanced in HCT15 cells by 70.23 ± 12.6% (Figure 4(b)). 36 hours of GLPG1790 treatment increased p53 expression levels in HCT116 but not in HCT15 cells (Figure 4(c)). The role of KRAS was investigated by assessing the effects of GLPG1790 on the activation status of ERK, which is known to be its principal pro-oncogenic downstream target. As shown in Figure 5(a), GLPG1790 (IC_50_) treatment persistently increased the ERK activation in HCT116 cells whilst no effects were observed in HCT15 cells (Figure 5(a)). Cotreating HCT116 cells with the MEK/ERK inhibitor U0126 (10 *μ*M) diminished the activity of *β*-galactosidase induced by GLPG1790 (Figure 5(b)) and similarly diminished the expression of the senescence marker proteins p16^INK4^, p21^Waf1/Cip1^, and p27^Cip/Kip^ (Figure 5(c)). Taken together, this evidence suggests that both p53 and KRAS-ERKs-mediated signaling mediate the antiproliferative, senescence-promoting effects of GLPG1790 differently depending on CRC cell type.

### 3.3. GLPG1790 *In Vivo* Reduces the Growth Capacity of CRC Cells

**Figure 6:**
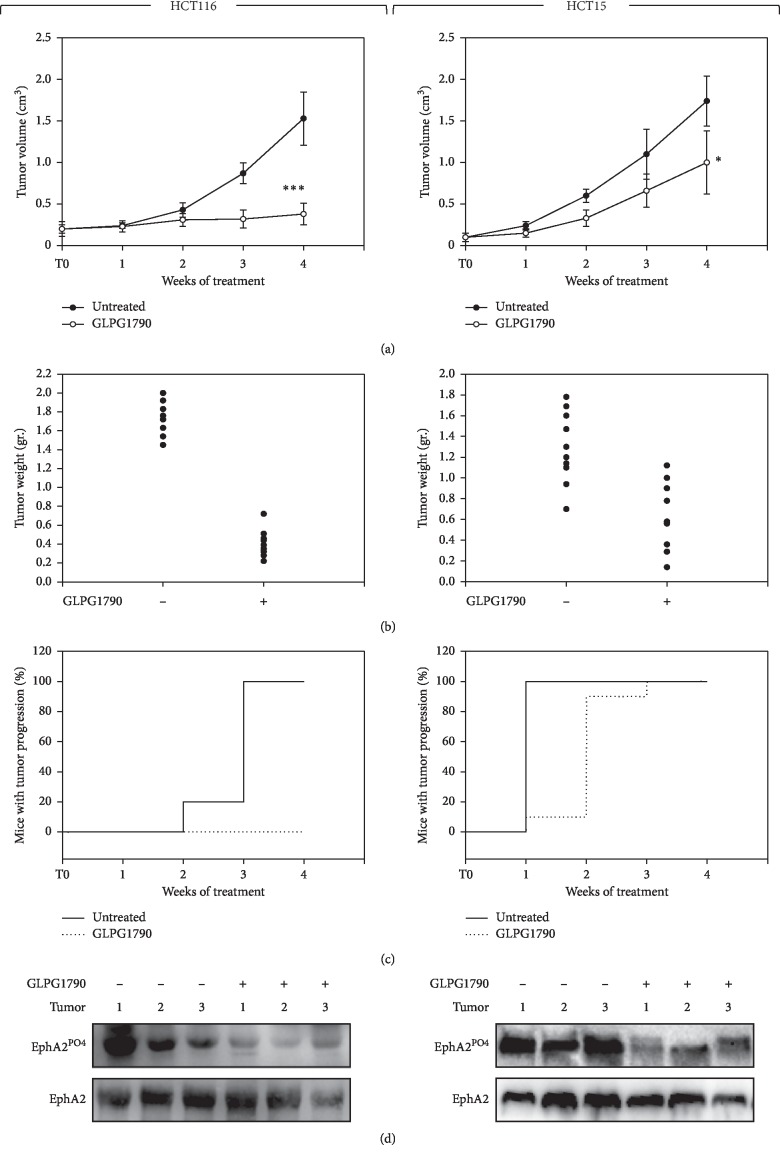
Effects of GLPG1790 on in vivo tumor growth. (a) Growth curves indicating tumor volume from xenografted HCT116 or HCT15 cell lines that have been untreated (vehicle) or GLPG1790-treated (GLPG1790) have been compared. Tumor volumes were evaluated as described in the methods section, and values represent the mean ± SEM from 10 mice. The upper panel shows the sequential treatments of xenografted mice, which began when tumors reached a volume of approximately 0.2–0.3 cm^3^. GLPG1790 (30 mg/kg) was administered 5 days a week for 4 weeks. (b) Tumor weights of mice that had remained untreated or had been treated with GLPG1790 have been shown. (c) Kaplan–Meier estimates for rates of progression of untreated and GLPG1790-treated HCT116-derived tumors. (d) Phosphorylation/activation status of EphA2 in tumors from vehicle- or GLPG1790-treated mice. A representative Western blot experiment from the 6 tumors analyzed has been shown.

Therapeutic efficiency of GLPG1790 was then investigated using a CRC xenograft tumor model in nude mice. For any cell line, twenty mice were subcutaneously injected with 1 × 10^6^ cells/per mouse; after tumors reached a volume of ∼0.2–0.3 cm^3^, animals were divided in two groups of 10 mice each. Animals were treated with GLPG1790 (30 mg/kg) 5 days/week for 4 weeks. As shown in Figure 6(a), at the end of the experiment, GLPG1790 reduced tumor volume by 74.6 ± 11.6% and 52.3 ± 11.2% in mice receiving HCT116 and HCT15 xenografts, respectively. Tumor weights were significantly decreased in mice treated with GLPG1790 in comparison to controls, with 70–90% inhibition of HCT116-derived tumors and 50–90% inhibition for HCT15-derived tumors (Figure 6(b)). The number of mice experiencing TP significantly differed across the groups, and this was confirmed by the median values of TP (Figure 6(c)). In the vehicle group, tumor progression was completed within three weeks of receiving HCT116 xenografts and within one week of receiving xenografts of HCT15 (Figure 6(c)). With respect to GLPG1790-treated mice, in HCT116-grafted mice, TP never occurred, while for HCT15-grafted mice, TP began in the first week and was completed two weeks later (Figure 6(c)). Immunoblotting on excised tumors showed that GLPG1790 treatment downregulated EPHA2 phosphorylation compared with tumors from vehicle-treated mice (Figure 6(d)).

### 3.4. GLPG1790 Affects the Expression of Epithelial-to-Mesenchymal Transition Markers and the Capacity of CRC Cells to Migrate

**Figure 7:**
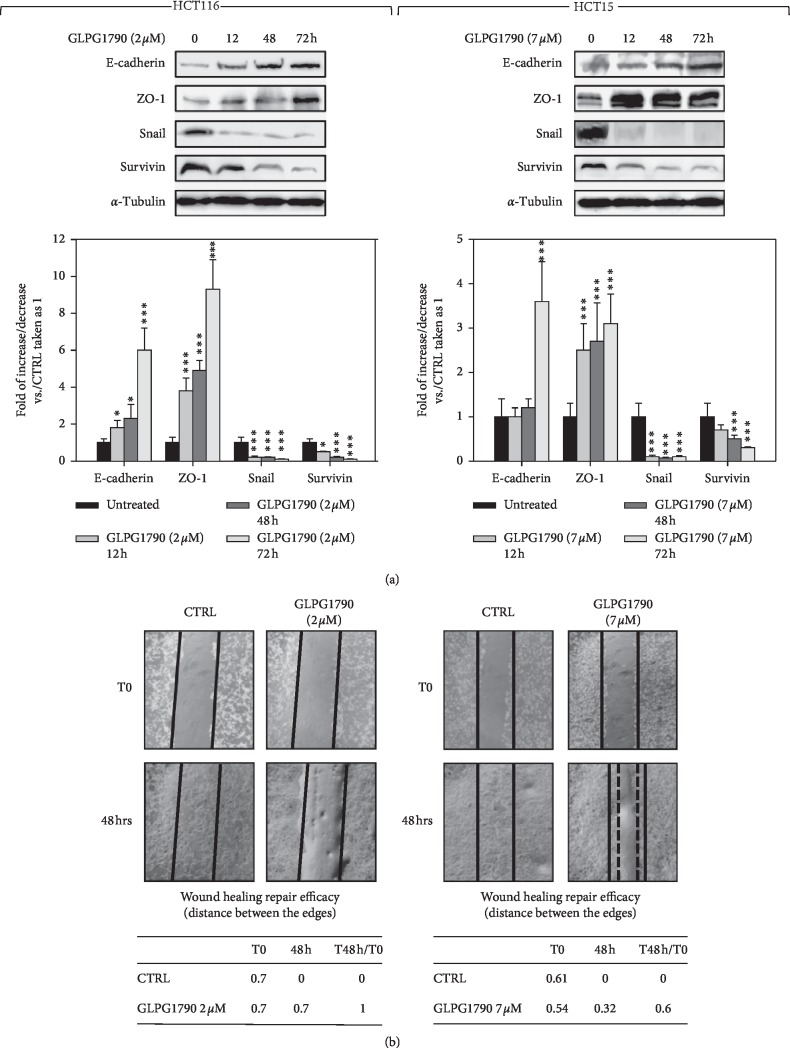
GLPG1790 diminishes the expression of epithelial-to-mesenchymal transition markers and impairs the ability of CRC cells to repair wounds. (a) Cell lysates from HCT116 and HCT15 cells ± GLPG1790 (IC_50_) at the indicated times were analyzed by immunoblotting with specific antibodies for indicated proteins. *α*-Tubulin expression indicates the loading of samples. Densitometric analysis of three independent experiments has been reported below the blots (^*∗*^*P* < 0.05, ^*∗∗*^*P* < 0.01, or ^*∗∗∗*^*P* < 0.001 vs. control). (b) Wound-healing experiments in HCT116 and HCT15 cells. A scratch was made at time 0 and maintained for 48 h in the presence of GLPG1790 (IC_50_) or DMSO. The dotted lines represent the edges of the wound. Photographs were taken under light microscope (10X magnification). The migration index (T48 h/T0) was reported in the table below the images.

The ability of GLPG1790 to delay the epithelial-to-mesenchymal transition (EMT) process and the related migration of CRC cells were investigated. GLPG1790 treatment (IC_50_) rapidly and persistently increased the expression of E-cadherin and ZO-1 and decreased levels of Survivin and Snail (Figure 7(a)). Further, the molecule efficiently reduced the capacity of both CRC cell lines to migrate (Figure 7(b)). Taken together, these data indicated that GLPG1790 restrains the pro-oncogenic EMT process and diminished the capacity of cancer cells to migrate.

## 4. Discussion

CRC remains an uncontrollable disease and is the second leading cause of cancer-related deaths [1]. As such, there is an urgent need for new therapeutic strategies to treat CRC. Eph, from the largest family of RTKs that by binding membrane bound ligands named ephrins, regulates many cellular functions through the modulation of several signal transduction pathways including the RAS/ERKs/MAPKs pathway [4, 5] known to sustain CRC [24]. CRC cells express high levels of different Eph proteins including EphA2, a known marker of poor prognosis in advanced CRC and a potentially critical therapeutic target for the treatment of CRC [7–9]. We have recently shown the anticancer proprieties of GLPG1790, a new pan inhibitor of the Eph receptors with a strong efficiency versus EphA2, versus several breast, rhabdomyosarcoma, and glioblastoma cell lines, both *in vitro* and *in vivo* [10–12]. Since our preliminary *in vitro* investigation showed the therapeutic potential of GLPG1790 also versus CRC [13], we decided to better characterize the pharmacological action of this drug on CRC by using *in vitro* and in *vivo* approaches on p53 wild-type or mutated CRC cell lines.

GLPG1790 decreased the phosphorylation/activation levels of EphA2 and affected CRC cell viability and proliferation, inducing the promotion of cell-cycle arrest in the G_1_/S phase of the cell cycle. According to the cell-cycle distribution, GLPG1790 downregulated cyclin E and PCNA, thus affecting strategic molecular targets for the onset and progression of CRC [25, 26]. Moreover, GLPG1790 upregulated the expression of cell-cycle inhibitors and tumor suppressors p21^Waf1/Cip1^ and p27^Cip/Kip^, which have previously been shown to revert the chemo-resistant phenotype of several cancer cell types [27]. Unexpectedly, GLPG1790 enhanced expression of cyclin D1, known to be a positive regulator of the G_1_/S cell-cycle transition [28]. We have recently shown that GLPG1790 upregulates the expression of cyclin D1 in RMS, inducing the cytoplasmatic/perinuclear accumulation of the protein [11]. Reports have shown that this accumulation correlates with a lower proliferative index in several cancer types [29, 30]. We suppose that a similar mechanism occurs in CRC cells, and future investigations will be to fully elucidate how the protein regulates proliferation. Notably, GLPG1790, at the concentration tested, did not induce apoptosis on CRC cell lines suggesting that it worked as a cytostatic pure treatment. However, GLPG1790 could sensitize cells to the cytotoxic action of other drugs, as some of our preliminary data [13] and ongoing experiments suggest.

Cytostatic drugs induce either reversible or irreversible cell-cycle arrest termed quiescence or senescence, respectively. During quiescence-related growth arrest, cells diminish metabolic activity, protein synthesis, and other cellular functions. This results in a lack cellular growth; however, removing cytostatic stimulus reactivates cell growth. Contrary to quiescence, cellular senescence is characterized by the nonreversible loss of proliferative potential, the increases in *β*-galactosidase activity, and cellular expansion [31–33]. GLPG1790 induced senescence in HCT116 cells as indicated by enhanced *β*-galactosidase activity and the acquisition of the characteristic morphology, which includes large and flattened cells that are markers of senescence [33]. Quiescence, however, occurred in the HCT15 cell line. The data collected here support the suggestion that GLPG1790 is a potentially powerful therapeutic with respect to the treatment of CRC. In fact, a treatment that induces senescence is expected to be more efficient than a treatment that induces quiescence [31]. This is because senescence is irreversible and also because senescent cells can be more easily eliminated via phagocytosis, a process that can result in tumor regression [32]. However, these data suggest that the ability of GLPG1790 to induce the maximum therapeutic effects against CRC progression likely depends on the molecular features of targeted cells.

The ability to induce quiescence or senescence depends on several signaling pathways, including p53 and RAS/ERKs/MAPKs [22, 23], known to drive the onset and progression of CRC [2, 3]. Though no relationship has previously been shown between the mutational status of either p53 or RAS/ERKs/MAPKs and Eph signaling dysregulation, we decided to investigate whether the proteins had roles in mediating GLPG1790-induced effects. Silencing p53 with siRNA specific for the protein enhanced the ability of GLPG1790 to induce senescence in HCT15 cells only, while having the opposite effect on HC116, suggesting that the role of p53 varies depending on cellular context. p53^WT^ and p53^MT^, expressed, respectively, by HCT116 and HCT15 [12], are known to promote or diminish, respectively, the activation of senescence in CRC [22, 34, 35]. This evidence seems to suggest that the mutational status of p53 could affect the ability of GLPG1790 to induce senescence and that the presence of the mutated p53, known to have acquired oncogenic features due to a gain-of-function mutation [36], has a negative effect on the therapeutic efficacy of GLPG1790. Furthermore, the fact that GLPG1790 increased the expression levels of p53 in HCT116 and did not induce any modulation in HCT15 cells reinforces the idea that p53^WT^ mediates whilst p53^MT^ counteracts senescence induced by the GLPG170. However, further tests will be needed to confirm whether there is indeed a relationship between the mutational status of p53 and the therapeutic efficiency of Eph inhibitors.

Approximately 30% to 50% of CRCs express a mutated RAS gene that promotes the constitutive activation of the ERK/MAPK downstream signaling pathway [37]. This pathway is a key regulator of cell proliferation and transformation, promotes CRC onset, and sustains CRC progression [38]. Interestingly, the Ephs are among the few receptor tyrosine kinases known to negatively regulate RAS signaling and its downstream pathways in a variety of cell types [39] though, under some circumstances, Eph receptors activate, rather than inhibit MAPK signaling [40]. We also have shown in previous reports that GLPG1790 induces irreversible growth arrest and differentiation by downregulating ERK in RMS cells [11]. Herein, GLPG1790 strongly and persistently increased the phosphorylation/activation of ERK signaling in HCT116 cells indicating that relationship between RAS and Eph signaling is similar to that of RMS cells. However, the fact that both RMS [40] and CRC [12] cell lines express constitutively activated RAS, which cannot be regulated, suggests that Eph could modulate the ERK activation downstream of Ras. This could also explain why GLPG1790 failed to modulate ERKs in HCT15 cells expressing constitutively active Ras. Interestingly, p53^WT^ has been shown to regulate ERK activation [41], and the presence of a mutated p53 in HCT15 could be the reason for the failure to activate ERKs after treatment with GLPG1790. Given the role of ERKs in promoting tumorigenesis, the fact that they are GLPG1790 activated in HCT116 might seem disadvantageous. However, depending on the duration, magnitude and its subcellular localization of ERKs activation various advantageous cell responses could be affected, such as proliferation, migration, differentiation, and death [42]. Though literature most often emphasizes the oncogenic potential of enhanced ERK activation, a growing number of conflicting studies have suggested that, under certain conditions, aberrant ERK activation can promote anticancer effects including senescence and cell death [43]. The biological significance of GLPG1790-mediated activation of ERKs requires further study; however, the fact that the inhibition of ERK signaling diminished senescence suggests an antioncogenic role for the protein.

CRC cells progressively acquire a metastatic phenotype. While several molecular mechanisms have been shown to orchestrate this process, the EMT has been shown to play a key role [44, 45]. EMT is a biologic process that allows a polarized epithelial cell to acquire a mesenchymal cell phenotype characterized by an enhanced migratory capacity and invasiveness [44, 45]. Considering that the Eph signaling primarily regulates the cell-to-cell interactions, we supposed that GLPG1790 could disrupt the EMT process by reducing the ability of CRC cells to migrate. Our hypothesis was confirmed by data collected here. Further, GLPG1790 more strongly disrupted EMT in HCT116 than in HCT15, in line with the role of p53 in contributing to metastasis [46].

Because of the natural limitations of the *in vitro* study model, we also tested the efficacy of the GLPG1790 in xenograft models. Herein, we have shown that although GLPG1790 efficiently blocked EphA2 phosphorylation in tumors derived from both HCT116- or HCT15-xenografted mice, HCT116 tumors were more sensitive to GLPG1790. These results were supported *in vitro* data and, taken together, suggest that the presence of p53^WT^ represents the best condition for use of Eph inhibition-based therapy for CRC.

The rate of both the incidence and mortality of CRC has declined over the last two decades. This success is attributable to the early detection and treatment of CRC [47]; however, CRC is still an uncontrollable disease and is the second leading cause of cancer-related deaths. The results of this study demonstrate the preclinical *in vitro* and *in vivo* antitumor activity of GLPG1790, a new potent pan inhibitor of EPH receptors, against human CRC cells. Data collected here further suggest that the mutational status of p53 could affect the responsiveness of CRC cells to therapy targeting Eph. Considering the fact that the CRC cell lines used here were derived from different tumors with completely different genetic backgrounds, other molecules may certainly participate in regulating responsiveness to GLPG1790 in CRC cells. These results indicating that targeting Eph signaling may be used in a signal transduction-based therapy for CRC warrant further testing in preclinical and clinical CRC trials.

## Data Availability

All data generated or analyzed during this study are included in this published article.

## References

[1] Jemal A., Bray F., Center M. M., Ferlay J., Ward E., Forman D. (2011). Global cancer statistics. *CA: A Cancer Journal for Clinicians*.

[2] Armaghany T., Wilson J. D., Chu Q., Mills G. (2012). Genetic alterations in colorectal cancer. *Gastrointest Cancer Res*.

[3] Fearon E. R., Vogelstein B. (1990). A genetic model for colorectal tumorigenesis. *Cell*.

[4] Kullander K., Klein R. (2002). Mechanisms and functions of Eph and ephrin signalling. *Nature Reviews Molecular Cell Biology*.

[5] Pasquale E. B. (2010). Eph receptors and ephrins in cancer: bidirectional signalling and beyond. *Nature Reviews Cancer*.

[6] Brantley-Sieders D., Schmidt S., Parker M., Chen J. (2004). Eph receptor tyrosine kinases in tumor and tumor microenvironment. *Current Pharmaceutical Design*.

[7] Boyd A. W., Bartlett P. F., Lackmann M. (2014). Therapeutic targeting of EPH receptors and their ligands. *Nature Reviews Drug Discovery*.

[8] Herath N. I., Boyd A. W. (2010). The role of Eph receptors and ephrin ligands in colorectal cancer. *International Journal of Cancer*.

[9] Dunne P. D., Dasgupta S., Blayney J. K. (2016). EphA2 expression is a key driver of migration and invasion and a poor prognostic marker in colorectal cancer. *Clinical Cancer Research*.

[10] Pujuguet P., Beirinckx F., Delachaume C. GLPG1790: the first ephrin (EPH) receptor tyrosine kinase inhibitor for the treatment of triple negative breast cancer.

[11] Megiorni F., Gravina G. L., Camero S. (2017). Pharmacological targeting of the ephrin receptor kinase signalling by GLPG1790 in vitro and in vivo reverts oncophenotype, induces myogenic differentiation and radiosensitizes embryonal rhabdomyosarcoma cells. *Journal of Hematology Oncology*.

[12] Gravina G. L., Mancini A., Colapietro A. (2019). The small molecule ephrin receptor inhibitor, GLPG1790, reduces renewal capabilities of cancer stem cells, showing anti-tumour efficacy on preclinical glioblastoma models. *Cancers*.

[13] Vitiello P. P., Mele L., Prisco C. (2019). GLPG 1790, a new selective EPHA2 inhibitor, is active in colorectal cancer cell lines belonging to the CMS4/mesenchymal-like subtype. *Annals of Oncology*.

[14] Ahmed D., Eide P. W., Eilertsen I. A. (2013). Epigenetic and genetic features of 24 colon cancer cell lines. *Oncogenesis*.

[15] Marampon F., Gravina G. L., Popov V. M. (2014). Close correlation between MEK/ERK and Aurora-B signaling pathways in sustaining tumorigenic potential and radioresistance of gynecological cancer cell lines. *International Journal of Oncology*.

[16] Marampon F., Gravina G. L., Festuccia C. (2016). Vitamin D protects endothelial cells from irradiation-induced senescence and apoptosis by modulating MAPK/SirT1 axis. *Journal of Endocrinological Investigation*.

[17] Gravina G. L., Mancini A., Ranieri G. (2013). Phenotypic characterization of human prostatic stromal cells in primary cultures derived from human tissue samples. *International Journal of Oncology*.

[18] Scicchitano B. M., Sorrentino S., Proietti G. (2018). Levetiracetam enhances the temozolomide effect on glioblastoma stem cell proliferation and apoptosis. *Cancer Cellular International*.

[19] Dobrowolny G., Martini M., Scicchitano B. M. (2018). Muscle expression of SOD1G93A triggers the dismantlement of neuromuscular junction via PKC-theta. *Antioxidants & Redox Signaling*.

[20] Pelosi L., Forcina L., Nicoletti C. (2017). Increased circulating levels of interleukin-6 induce perturbation in redox-regulated signaling cascades in muscle of dystrophic mice. *Oxidative Medicine and Cellular Longevity*.

[21] Sciandra F., Scicchitano B. M., Signorino G. (2017). Evaluation of the effect of a floxed Neo cassette within the dystroglycan (Dag1) gene. *BMC Research Notes*.

[22] Kang J.-Y., Kim J. J., Jang S. Y., Bae Y.-S. (2009). The p53-p21(Cip1/WAF1) pathway is necessary for cellular senescence induced by the inhibition of protein kinase CKII in human colon cancer cells. *Molecules and Cells*.

[23] Dimauro T., David G. (2010). Ras-induced senescence and its physiological relevance in cancer. *Current Cancer Drug Targets*.

[24] Saeed O., Lopez-Beltran A., Fisher K. W. (2019). RAS genes in colorectal carcinoma: pathogenesis, testing guidelines and treatment implications. *Journal of Clinical Pathology*.

[25] Kitahara K., Yasui W., Kuniyasu H. (1995). Concurrent amplification of cyclin E and CDK2 genes in colorectal carcinomas. *International Journal of Cancer*.

[26] Choi H. J., Jung I. K., Kim S. S., Hong S. H. (1997). Proliferating cell nuclear antigen expression and its relationship to malignancy potential in invasive colorectal carcinomas. *Diseases of the Colon & Rectum*.

[27] Abukhdeir A. M., Park B. H. (2008). P21 and p27: roles in carcinogenesis and drug resistance. *Expert Reviews in Molecular Medicine*.

[28] Fu M., Wang C., Li Z., Sakamaki T., Pestell R. G. (2004). Minireview: cyclin D1: normal and abnormal functions. *Endocrinology*.

[29] Palmqvist R., Stenling R., Öberg Å., Landberg G. (1998). Expression of cyclin D1 and retinoblastoma protein in colorectal cancer. *European Journal of Cancer*.

[30] Comstock C. E. S., Revelo M. P., Buncher C. R., Knudsen K. E. (2007). Impact of differential cyclin D1 expression and localisation in prostate cancer. *British Journal of Cancer*.

[31] Terzi M. Y., Izmirli M., Gogebakan B. (2016). The cell fate: senescence or quiescence. *Molecular Biology Reports*.

[32] Xue W., Zender L., Miething C. (2007). Senescence and tumour clearance is triggered by p53 restoration in murine liver carcinomas. *Nature*.

[33] Carnero A. (2013). Markers of cellular senescence. *Methods in Molecular Biology*.

[34] Vogelstein B., Lane D., Levine A. J. (2000). Surfing the p53 network. *Nature*.

[35] Qian Y., Chen X. (2013). Senescence regulation by the p53 protein family. *Methods in Molecular Biology*.

[36] Bossi G., Marampon F., Maor-Aloni R. (2008). Conditional RNA interference in vivo to study mutant p53 oncogenic gain of function on tumor malignancy. *Cell Cycle*.

[37] AACR Project GENIE Consortium (2017). AACR project GENIE: powering precision medicine through an international consortium. *Cancer Discovery*.

[38] Tai C. J., Chang C. C., Jiang M. C. (2012). Clinical-pathological correlation of K-Ras mutation and ERK phosphorylation in colorectal cancer. *Polish Journal of Pathology*.

[39] Noren N. K., Pasquale E. B. (2004). Eph receptor-ephrin bidirectional signals that target Ras and Rho proteins. *Cellular Signalling*.

[40] Pratt R. L., Kinch M. S. (2002). Activation of the EphA2 tyrosine kinase stimulates the MAP/ERK kinase signaling cascade. *Oncogene*.

[41] Singh S., Upadhyay A. K., Ajay A. K., Bhat M. K. (2007). p53 regulates ERK activation in carboplatin induced apoptosis in cervical carcinoma: a novel target of p53 in apoptosis. *FEBS Letters*.

[42] Murphy L. O., Blenis J. (2006). MAPK signal specificity: the right place at the right time. *Trends in Biochemical Sciences*.

[43] Cagnol S., Chambard J.-C. (2010). ERK and cell death: mechanisms of ERK-induced cell death—apoptosis, autophagy and senescence. *FEBS Journal*.

[44] Spaderna S., Schmalhofer O., Hlubek F. (2006). A transient, EMT-linked loss of basement membranes indicates metastasis and poor survival in colorectal cancer. *Gastroenterology*.

[45] Tsai J. H., Yang J. (2013). Epithelial-mesenchymal plasticity in carcinoma metastasis. *Genes & Development*.

[46] Powell E., Piwnica-Worms D., Piwnica-Worms H. (2014). Contribution of p53 to metastasis. *Cancer Discovery*.

[47] Gupta A. K., Brenner D. E., Turgeon D. K. (2008). Early detection of colon cancer: new tests on the horizon. *Molecular Diagnosis & Therapy*.

